# Role of proprioceptors in chronic musculoskeletal pain

**DOI:** 10.1113/EP090989

**Published:** 2023-07-07

**Authors:** Cheng‐Han Lee, Chih‐Cheng Chen

**Affiliations:** ^1^ Institute of Biomedical Sciences Academia Sinica Taipei Taiwan; ^2^ Neuroscience Program of Academia Sinica Academia Sinica Taipei Taiwan; ^3^ Taiwan Mouse Clinic, Biomedical Translational Research Center Academia Sinica Taipei Taiwan

**Keywords:** chronic musculoskeletal pain, glutamate, lactate, muscle spindle, proprioceptors, substance P, tissue acidosis

## Abstract

Proprioceptors are non‐nociceptive low‐threshold mechanoreceptors. However, recent studies have shown that proprioceptors are acid‐sensitive and express a variety of proton‐sensing ion channels and receptors. Accordingly, although proprioceptors are commonly known as mechanosensing neurons that monitor muscle contraction status and body position, they may have a role in the development of pain associated with tissue acidosis. In clinical practice, proprioception training is beneficial for pain relief. Here we summarize the current evidence to sketch a different role of proprioceptors in ‘non‐nociceptive pain’ with a focus on their acid‐sensing properties.

## Abstract (highlights)

- **What is the topic of this review?**
  Why proprioceptors, which are non‐nociceptive, low‐threshold mechanosensory neurons that monitor muscle contraction and body position, express several proton‐sensing ion channels and receptors.
- **What advances does it highlight?**
  ASIC3 is a dual function protein for proton‐sensing and mechano‐sensing in proprioceptors that can be activated by eccentric muscle contraction or lactic acidosis. A role is proposed for proprioceptors in non‐nociceptive unpleasantness (or sng), which is associated with their acid‐sensing properties, in chronic musculoskeletal pain.

## 1 INTRODUCTION

**FIGURE 1:**
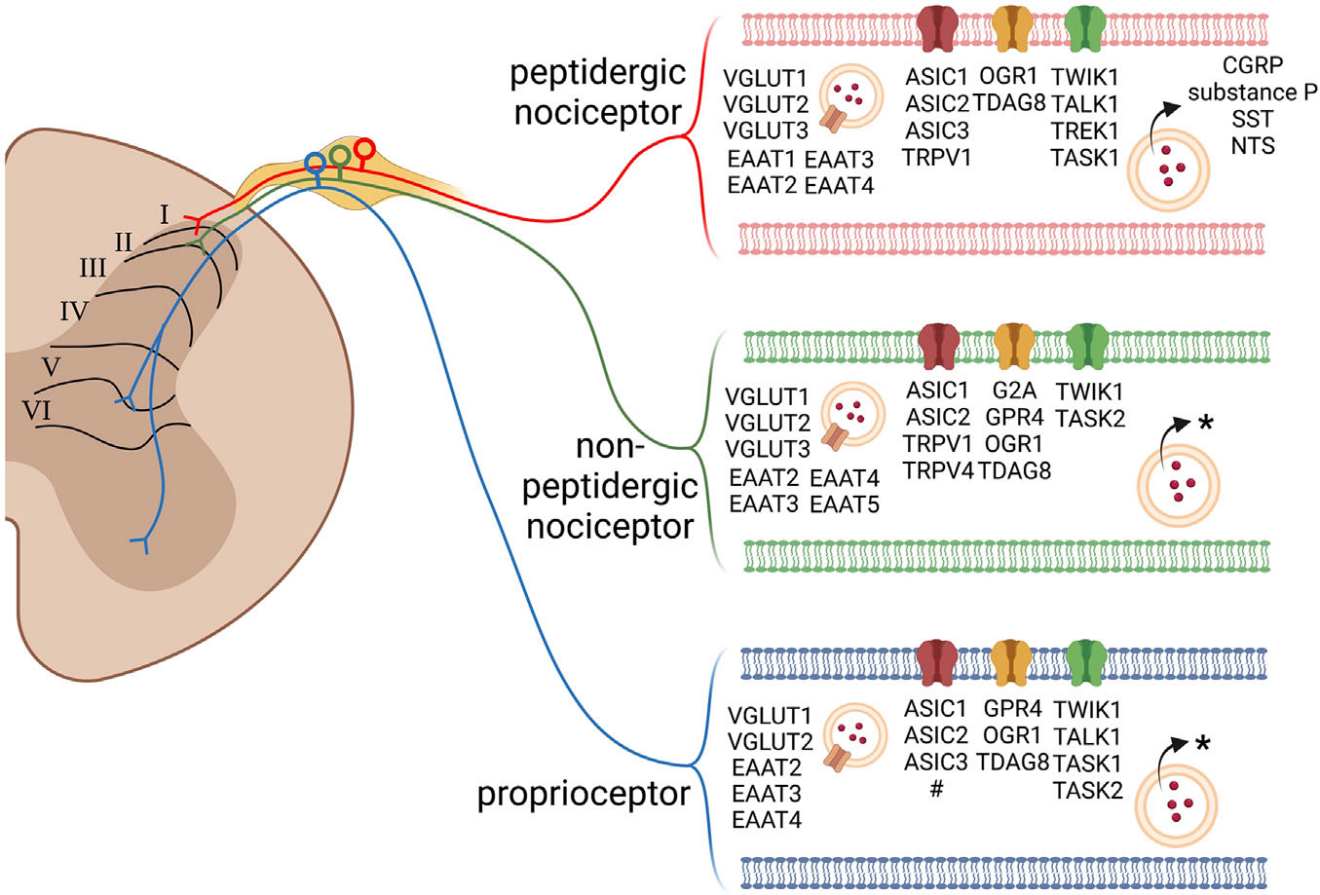
Transcriptomic profiles of proton‐sensing channels and receptors, neuropeptides and vesicle glutamate transporters in three major sub‐populations of dorsal root ganglion neurons. From single‐cell transcriptomic analyses, proton‐sensing channels and receptors (ASICs, TRPs, K2Ps, GPCRs), neuropeptides (e.g., substance P, CGRP) and vesicle glutamate transporters (vGlutRs, EAATs) were found differentially expressed in peptidergic nociceptors (innervated in lamina I of the spinal cord), non‐peptidergic nociceptors (innervated in inner lamina II of the spinal cord) and proprioceptors (innervated in lamina V and VI of the spinal cord). *Some neuropeptides were identified to a lesser extent in non‐peptidergic nociceptors and proprioceptors than peptidergic nociceptors. #TRPV1 was identified in a small population of proprioceptors.

Tissue acidosis is a major risk factor in the development of chronic musculoskeletal pain (Hung et al., 2023; Lin et al., 2018). Accumulating evidence has shown that proton‐sensing ion channels (acid‐sensing ion channels (ASICs), transient receptor potential (TRP) channels, two‐pore potassium (K2P) channels) and G‐protein‐coupled receptors (G2A, GPR4, OGR1, TDAG8) are involved in pain associated with tissue acidosis (Lin et al., 2018). Notably, proton‐sensing ion channels/receptors are expressed in nociceptors as well as in non‐nociceptors such as proprioceptors (Figure 1, Table 1) (Lin et al., 2016; Nakamura & Jang, 2014; Usoskin et al., 2015). Why proprioceptive afferents need the acid‐sensing property remains unknown. In addition, although non‐nociceptive cutaneous afferents could contribute to pain hypersensitivity in chronic pain states, whether non‐nociceptive muscle afferents are also involved in pain hypersensitivity of deep tissues is unclear. In 1996, Gillette and colleagues reviewed clinical reports and hypothesized a potential role for proprioceptive afferents in producing ‘non‐nociceptive pain’ associated with peripheral and central neuropathy, fibromyalgia, trauma‐induced pain, idiopathic low back pain and chronic regional pain syndrome (Kramis et al., 1996).

**TABLE 1:** RNAseq results for the mRNA expression of acid‐sensing ion channels/receptors in proprioceptors.

	Subtype of proprioceptor			Pv^+^ cluster
	de Nooij (*n* = 242)	Lallemend (*n* = 1109)	Ernfors (*n* = 622)	Woolf (*n* = 92)
ASIC1	C1–C5	Ia 1–3, Ib, II 1–4 (high)	NF4, 5 (high)	Low
ASIC2	C1–5	Ia 1–3, Ib, II 1–4 (high)	NF4, 5 (few)	High
ASIC3	C5 (high), C1–4 (few)	Ib, II 4 (high); II 1–3, Ia 1–3 (low)	NF4 (medium)	High
TRPV1	C1, C4 (few)	Ia 2, Ib, II 1–3 (few)	NF4 (few)	Few
TRPV4	C3 (few)	Ia 1–3, Ib, II 2–4 (few)		Few
TRPA3				Few
G2A				
GPR4	C3 (few)	Ia 1‐2, II 2 (few)	NF4 (few)	
OGR1		Ia 1–3, Ib, II 1–4 (high)	NF4, 5 (few)	Few
TDAG8			NF5 (few)	
TASK1	C1–C5	II 4 (high); Ia 1–3, Ib, II 1–3 (medium)	NF5 (few)	Low
TASK2		Ia 2, II 3 (few)	NF4 (few)	
TASK3		Ia 1, II 1 (few)		Few
TALK1			NF5 (few)	
TREK1	C1, C3, C4, C5 (few)	Ia 2, II 2‐4 (low); Ib (few); II 1 (medium)		Few
TWIK1	C1–C5	Ia 1–3 (high); Ib, II 1–3 (medium)	NF4, 5 (high)	High
Reference	Oliver et al. (2021)	Wu et al. (2021)	Usoskin et al. (2015)	Chiu et al. (2014)

mRNA expression profiles of acid‐sensing ion channels, transient receptor potential channels, acid‐sensing G‐protein‐coupled receptors and two‐pore potassium channels in proprioceptors from four RNAseq datasets. C1: type Ia afferent; C2–4: type II afferent; C5: type Ib afferent. Ia 1–3: three subtypes of type Ia afferents; II 1–4: four subtypes of type II afferents. High, medium, low and few represent transcriptome level or cell number in Pv^+^ neurons. Pv, parvalbumin.

In this review, we explore some possible roles of non‐nociceptive afferents (e.g., proprioceptors) in chronic musculoskeletal pain.

## 2 ACID SIGNALING IN NOCICEPTIVE AND NON‐NOCICEPTIVE SOMATOSENSORY NEURONS

### 2.1 Promiscuous nature of acid sensation

In response to tissue acidosis, somatosensory neurons express various proton‐sensing ion channels and receptors, including ASICs, TRP channels, K2P channels and proton‐sensing G‐protein‐coupled receptors (Lin et al., 2018). Single‐cell transcriptomic analyses demonstrated that proton‐sensing ion channels and receptors are differentially expressed in three major somatosensory neuron populations: peptidergic nociceptors, non‐peptidergic nociceptors and proprioceptors (Figure 1) (Usoskin et al., 2015). From psychophysical studies of healthy volunteers, ASICs have become known as the major acid‐sensors for acid‐induced pain in human nociceptors (Jones et al., 2004; Ugawa et al., 2002). In animal studies, ASICs were found to play important roles in acute or chronic pain mechanisms, including delayed onset muscle soreness (DOMS), inflammatory, neuropathic, and chemical‐injury induced pain, and fibromyalgia models (Chang et al., 2019; Chen et al., 2002, 2014; Deval et al., 2008; Diochot et al., 2016; Fujii et al., 2008; Matsubara et al., 2019; Sluka et al., 2003, 2013; Yen et al., 2009). Paradoxically, the pro‐nociceptive ASICs are expressed in non‐nociceptive proprioceptors, contributing to proprioceptive functions (Lin et al., 2016), and in muscle afferent neurons that mediate antinociceptive signalling (Chen & Chen, 2014; Han et al., 2019, 2022). Thus, acid sensation is a result of signalling integration between different somatosensory neurons including nociceptors, proprioceptors and other neurons.

### 2.2 Acid signalling in muscle nociception

In a mouse model of fibromyalgia, repeated injections of pH 4.0 acidic saline to the unilateral gastrocnemius muscle induced bilateral, long‐lasting mechanical hyperalgesia of muscle and hindpaws (Sluka et al., 2003). ASIC1b, ASIC3 and TRP channel subfamily V member 1 (TRPV1) were found to be the molecular determinants of acid‐induced fibromyalgia‐like pain in mice (Chang et al., 2019; Chen et al., 2014). Similarly, ASIC3 was found to be the major molecular determinant in two other fibromyalgia pain models induced by intermittent cold stress or repeated and intermittent sound stress (Hsu et al., 2019; Hung et al., 2020).

In other muscle pain models, both pharmacological and genetic approaches revealed that TRP channels and ASICs, especially ASIC3, contribute to the development of DOMS‐induced mechanical hyperalgesia (Fujii et al., 2008; Matsubara et al., 2019). In inflammatory muscle pain studies, amiloride, a pan‐ASIC blocker, could suppress carrageenan‐induced inflammatory muscle pain (Fujii et al., 2008). Hence, although ASIC1b, ASIC3 and TRPV4 are major pro‐nociceptive acid‐sensing molecules involved in the development of chronic muscle hyperalgesia, how different muscle afferents contribute to and integrate acid signalling is unknown.

### 2.3 Anti‐nociceptive acid signalling in muscle

**FIGURE 2:**
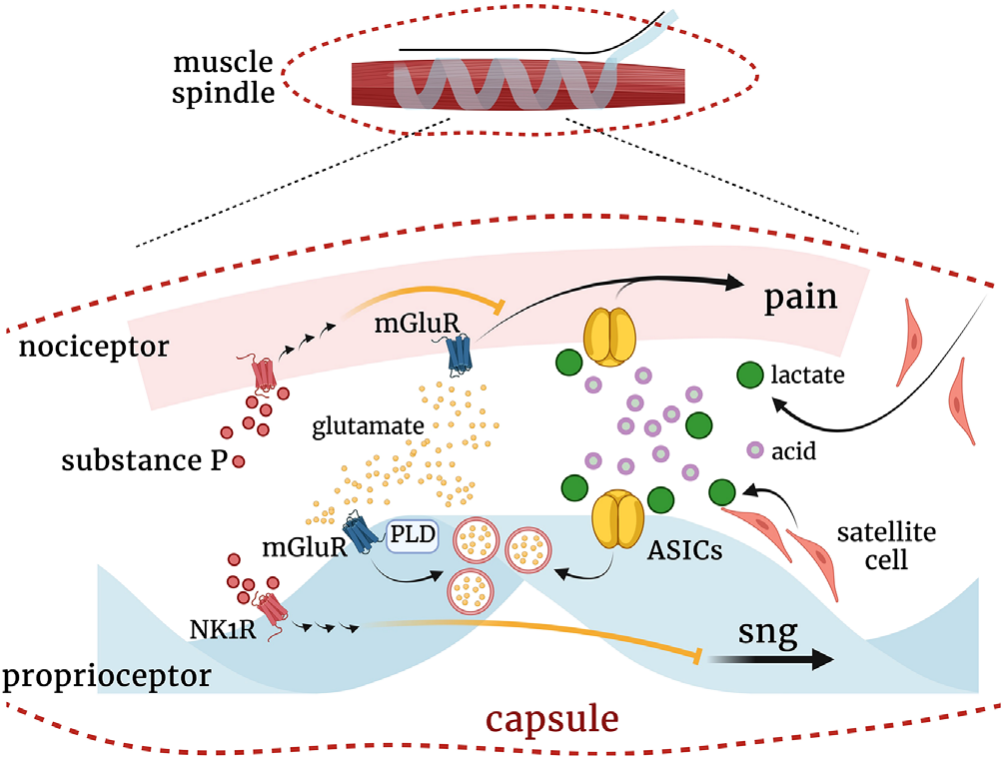
A schematic model of chronic nociception induced by hyperactive proprioceptors. With capsule leakage and altered satellite cell metabolic profile in microdamaged proprioceptors, lactate concentration is increased to create a local acidic environment. Proprioceptors become hyperactive via local acidosis by activating ASICs on proprioceptors, which leads to glutamate release. Nociceptors are activated by glutamate, and the glutamate release becomes a vicious cycle in the capsule. Hyperactive proprioceptors trigger an unpleasant feeling of sng. mGluR, metabotropic glutamate receptor; PLD, phospholipase D.

Of note, when both ASIC3 and TRPV1 are inhibited in the intramuscular acid injection regime, the remaining acid signalling, which is independent of ASIC3 and TRPV1, can trigger a prolonged anti‐nociceptive effect lasting for 48 h (Chen & Chen, 2014). The acid‐mediated antinociception requires the release of a neuropeptide, substance P, from muscle afferents (Figure 2). In most nociceptor subtypes (e.g., cutaneous peptidergic nociceptors), substance P is a well‐known pain neurotransmitter that works together with glutamate in the spinal cord to facilitate central sensitization and mediates neurogenic inflammation in the periphery to trigger peripheral sensitization (Chang et al., 2019). However, substance P can mediate antinociceptive signalling in muscle afferents, where it acts on neurokinin 1 receptors via a G‐protein‐independent, tyrosine kinase‐dependent pathway to activate Kv7 channels and hyperpolarize neurons (Lin et al., 2012).

Previous studies have shown that pH 5.0 acidic buffer can induce an inward current in 85% of muscle‐afferent dorsal root ganglion neurons, and ∼60% of these acid‐sensitive neurons express an acid‐induced current independent of ASIC3 and TRPV1 (Chen et al., 2014). Although the molecular identity of the ASIC3‐, TRPV1‐independent acid sensors is unknown, a possible candidate is ASIC1a, because ASIC1a can mediate anti‐nociceptive signalling in muscle afferents in response to dextrose prolotherapy in the acid‐induced fibromyalgia model (Han et al., 2022). ASIC1a‐mediated anti‐nociception also depends on the release of substance P from muscle afferents. The above studies reveal that most acid‐sensitive muscle afferents are not nociceptive and some are even anti‐nociceptive. Echoing this observation, recent RNAseq studies have shown that ASICs and other proton‐sensing ion channels/receptors are largely expressed in non‐nociceptive proprioceptors (Chiu et al., 2014; Oliver et al., 2021; Usoskin et al., 2015; Wu et al., 2021) (Tables 1 and 2).

**TABLE 2:** Association of transcriptomic clusters among publications.

Target gene	Ernfors (Usoskin et al., 2015)	de Nooji (Oliver et al., 2021)	Lallemend (Wu et al., 2021)
*Heg1*	NF5 (low)	C1, 2, 3, 4	Ia1, 2, II2
*Hpse*	NF5 (low)	C1	Ia2, 3
*Colq*	NF5 (medium)	C1	Ia2, 3
*Agpat4*	NF5 (few)	C1	Ia2, 3
*Calb2*	NF5 (low)	C1	Ia3
*Nxph1*	NF4, 5 (medium)	C2, 3, 4	II1, 2
*Fam196a*	NF5 (few)	C2, C4 (few)	
*Cdh13*	NF4, 5 (low)	C4	II1, 2
*Pcdh8*	NF4 (low), NF5 (medium)	C2, 3, 4 (few), C5	Ib, II4
*Tac1*	NF4 (low), NF5 (few)	C4	II1
*Itga2*	non‐NF4, 5	C5	Ib
*Chad*	NF5 (low)	C5	Ib, II4
*Pcdh17*	NF4 (low)	C5	Ib, II3

In the target gene column, all genes are classified by De Nooji's group to serve as representatives for proprioceptor classification.

### 2.4 ASICs in proprioceptors

Intriguingly, proprioceptors highly express the pro‐nociceptive ASIC3, which is a molecular determinant involved in the acid‐induced pain model and pain chronicity in two other mouse models of fibromyalgia induced by intermittent cold stress or repeated and intermittent sound stress (Hsu et al., 2019; Hung et al., 2020; Sluka et al., 2003). Although we previously showed that ASIC3 is a dual‐function protein involved in both acid sensation and tether‐mode mechanotransduction, the acid‐sensing role of proprioceptors is still a mystery (Lin et al., 2016). Besides ASIC3, proprioceptors also express many other proton‐sensing ion channels/receptors such as ASIC1, ASIC2, TRPV1, TRPV4, TASK1, TREK1, TWIK1 and GPR4 (Figure 1 and Table 1). Whether the acid‐sensitive proprioceptors have a possible pro‐nociceptive role remains to be investigated.

## 3 PROPRIOCEPTORS AND PAIN

Proprioceptors are groups of non‐nociceptive, low‐threshold mechanosensory neurons projecting to muscle spindles and Golgi tendon organs, where they monitor the status of muscle contraction and body position (Kroger, 2018). Since 1954, accumulated clinical evidence has shown a proprioceptive deficit among patients with chronic pain such as chronic low back pain and fibromyalgia (Contreras, 1954; Gucmen et al., 2022; Koumantakis et al., 2002; Nielsen et al., 1987; Peng et al., 2021). With a lesion of the central nervous system, central pain syndrome is most frequently associated with mild to severe disruption of the anterolateral ascending system with partial or complete preservation of the dorsal column/medial lemniscus function, which suggests the possible involvement of proprioceptive inputs (Beric, 1993). An interesting observation to support a role for proprioceptors in pain is that vibration (80 Hz) of an unexercised muscle is painless, whereas after eccentric exercise, the same vibration becomes painful (Roll et al., 1989; Weerakkody, Percival, et al., 2003). The exact source of the pain remains unclear, but an obvious candidate is the muscle spindle. Hence, we need to understand the role of proprioceptors in chronic musculoskeletal pain to further manipulate the proprioceptive input for relieving proprioception‐related unpleasantness.

Anatomically, terminals of proprioceptive afferents innervating muscle spindles and Golgi tendon organs are covered by a capsule to prevent the diffusion of extrafusal substances into the intrafusal space. Some capsules feature innervation of nociceptive terminals (Lund et al., 2010). Therefore, patients with musculoskeletal pain might have an altered microenvironment in the capsule (Partanen, 2018). From studies of DOMS, we could consider two hypotheses for the development of chronic muscle soreness, that an altered microenvironment in capsules includes (1) microdamage of proprioceptor terminals and (2) lactate and acidosis, both proposed to contribute to the development of muscle pain (Figure 2).

### 3.1 Microdamage of proprioceptors

Since 1995, accumulated evidence has demonstrated that proprioception can be disturbed by both eccentric and concentric exercise (Allen et al., 2007; Brockett et al., 1997; Proske, 2019; Proske & Gandevia, 2012; Saxton et al., 1995; Sonkodi et al., 2022; Weerakkody, Percival, et al., 2003). Sonkodi et al. (2020) proposed that DOMS is an acute compression axonopathy. Previous studies of DOMS suggested that overstretching from accelerating/eccentric movement leads to microdamaged local tissues, including proprioceptive terminals, and impairs proprioception function because the energy cannot be absorbed by muscles and other tissues (Friden & Lieber, 1992; Saxton et al., 1995; Sonkodi et al., 2022). The microdamage could include damage of neuronal membrane and/or an abnormal annulospiral structure of proprioceptive terminals. Patients with chronic musculoskeletal pain often exhibit proprioception deficits, which might be associated with functional abnormality or impaired proprioceptive neural terminals. Like axon reflex activity in nociceptor terminals, proprioceptor terminals can release neurotransmitters in response to stimuli. Anatomically, proprioceptor terminals contain synaptic‐like vesicles and dense core vesicles, which may store small‐molecule neurotransmitters and neuropeptides, respectively (Figure 1) (Bewick et al., 2005). Previous studies demonstrated that muscle stretch can trigger glutamate release from proprioceptor terminals and that glutamate can further potentiate proprioceptor afferent firing and possibly activate surrounding nociceptive afferents (Bewick & Banks, 2015). Accordingly, when proprioceptor terminals are injured, they would trigger the neurogenic effect via glutamate release, which thus results in a glutamate vicious cycle to hyperexcite the nerves (Figure 2). Moreover, tissue injury induces local inflammation. Hence, in muscle spindles, microdamage of proprioceptor terminals would trigger a neurogenic effect via glutamate release and induce local inflammation to activate nociceptors, which are expressed near proprioceptor terminals in capsules (Lund et al., 2010).

However, some studies did not support the exercise‐induced microdamage hypothesis. Gregory and colleagues found nearly normal muscle spindle responses to stretch and fusimotor stimulation in a muscle that had been subjected to a number of eccentric contractions sufficient to drop the muscle force by 46% (Gregory et al., 2004). This finding suggests that intrafusal muscle fibres are less susceptible to damage than are extrafusal muscle fibres after eccentric exercise. Further studies are needed to elaborate the effect of eccentric exercise on proprioceptor terminals.

### 3.2 Role of lactate

Intrafusal satellite cells are a group of astrocyte‐like cells similar to astrocytes in the central nervous system that play important roles in nutrition support, metabolic switch, structure maintenance and neuronal plasticity (Sonkodi, 2022). Intrafusal satellite cells also contribute to the increased permeability of the selective barrier of the muscle spindle (Sonkodi et al., 2020). A subpopulation of satellite cells, named quiescent muscle satellite cells, which have a lower metabolic rate and fewer active mitochondria than most satellite cells in normal muscle, may promote glycolysis (Iepsen et al., 2022; Montarras et al., 2013; Robergs et al., 2004). Furthermore, the astrocyte–neuron lactate shuttle (ANLS) hypothesis has been proposed in the brain, with astrocytes increasing glucose uptake, glycolysis and lactate release in response to hyperexcited neurons (Mason, 2017). ANLS metabolic machinery may also exist between intrafusal satellite cells and proprioceptor terminals (Figure 2). Although proprioceptor terminals are hyperexcited by abnormal stress via local glutamate signalling, the metabolism of intrafusal satellite cells would increase lactate release in response to nerve hyperexcitability in muscle spindles. Hence, lactate concentrations would increase. In clinical studies, microdialysis analyses of metabolomic profiles in interstitial or fascia revealed increased levels of glutamate and lactate under resting, low‐force exercise or repetitive work in patients with chronic muscle pain, such as fibromyalgia, chronic shoulder pain and chronic trapezius pain (Gerdle et al., 2010; Gerdle, Ghafouri, et al., 2014; Gerdle, Larsson, et al., 2014; Larsson et al., 2008; Malatji et al., 2017; Sorensen et al., 2018). Of note, the increased lactate levels in patients with chronic muscle pain were in the range of 1.5–6.5 mM, which are lower than the levels (15 mM) increasing blood pressure and heart rates (Gregory et al., 1987). Lactate is a potent activator of ASICs, which have high expression in a variety of peripheral sensory neurons, including proprioceptors (Immke & McCleskey, 2001; Oliver et al., 2021). Thus, proprioceptors would become hyperactive by being immersed in acidotic and high levels of glutamate and the lactate environment in muscle spindles via activating ASIC3 and inducing a glutamate vicious cycle (Figure 2). Abnormal activities of proprioceptors would cause poor coordination of proprioception and possibly induce nociceptor activation and the development of proprioceptive allodynia.

### 3.3 A pro‐nociceptive role for proprioceptors

Previous reports demonstrated that the nerve terminals of proprioceptors contain glutamate vesicles and some dense vesicles (possibly containing neuropeptides), which would be released when proprioceptors are stretched (Bewick & Banks, 2015). Also, the vesicular glutamate transporter is expressed in proprioceptor terminals in the masseter muscle (Lund et al., 2010). Therefore, proprioceptors may contribute to the development of chronic muscle pain mediated by ASIC‐induced glutamate release during local tissue acidosis.

## 4 CLINICAL IMPLICATIONS

### 4.1 Effect of exercise and proprioception training on pain relief

**FIGURE 3:**
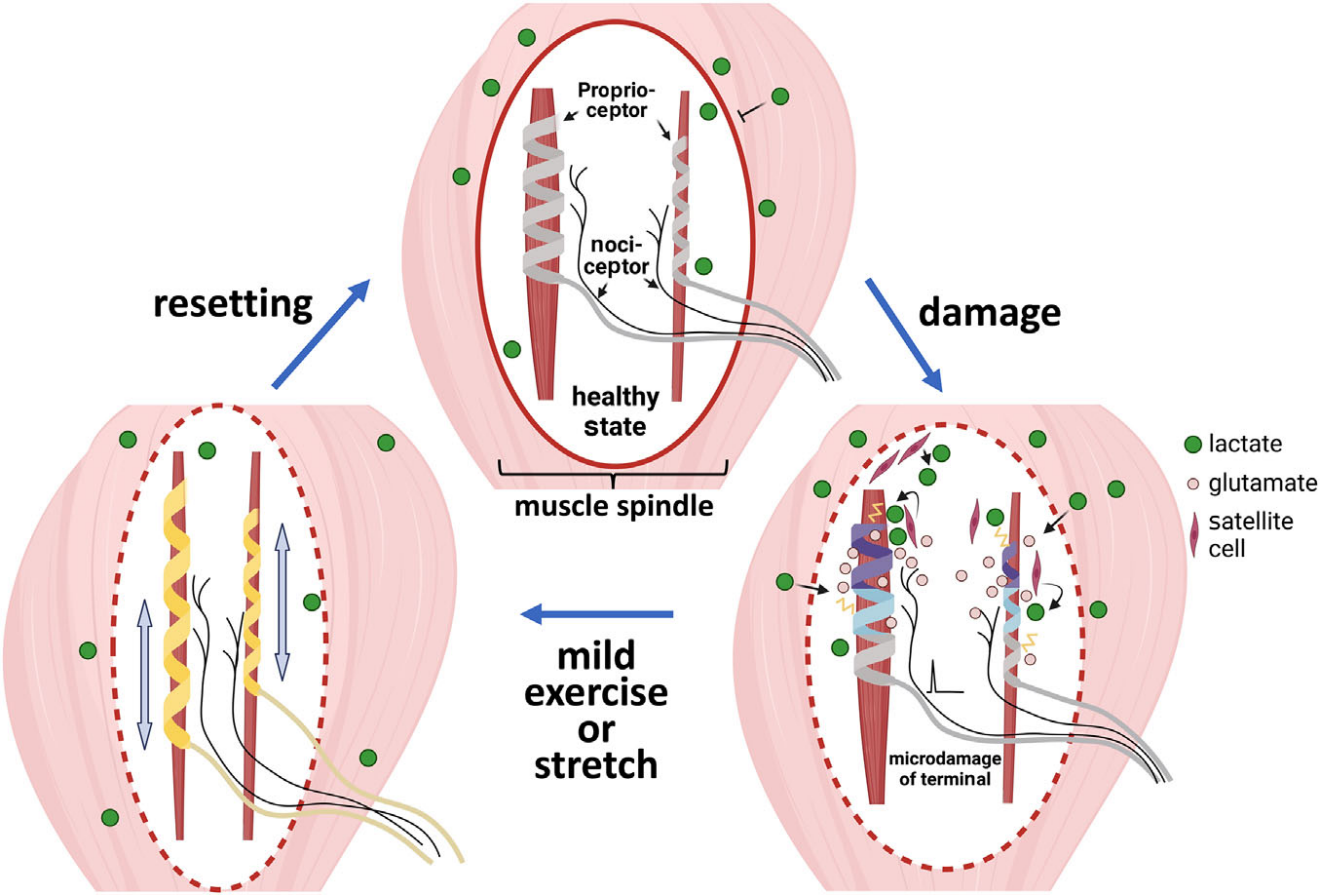
A schematic model of involvement of proprioceptors in chronic pain and resetting of proprioceptor activities by proprioception‐improving exercises. In the healthy state, the muscle spindle is covered in a tight capsule to control the micro‐environment for normal function. With capsule leakage due to proprioceptor microdamage, lactate permeability is increased, and satellite cells alter their metabolic profile to increase lactate release. Lactate activates ASICs on the membrane of proprioceptors in the capsule. Furthermore, microdamaged proprioceptors become hyperactive and activate nociceptors via glutamate release. Proprioception‐improving exercises, such as proprioceptive neuromuscular facilitation and Tai Chi, stretch the muscle spindle and alter the state of proprioceptors without lactate over‐production and reset the activities of proprioceptors to normal.

Physical therapy can introduce many non‐invasive ways to relieve pain in patients with chronic muscle pain. One non‐invasive way is exercise. From 1994, many clinical reports have demonstrated that exercise (e.g., proprioceptive neuromuscular facilitation (PNF), Tai‐Chi) can decrease pain and even improve the quality of life in people with different illnesses such as fibromyalgia, osteoarthritis, neck pain and chronic low back pain (Table 2). All these types of exercise can improve muscle strength and flexibility and also enhance proprioceptive function. Theoretically, many factors could work together to improve the proprioception deficits and pain, such as increased blood flow to maintain oxygen and a nutrient state, remove waste and increase the healing rate of the injured tissues. From a biomechanical view, as compared with resting, these exercise‐associated physical therapies actively lengthen the muscle and tendon under a non‐harmful force. We hypothesize these treatments might fine‐tune the annulospiral structure of type Ia and II proprioceptor terminals and the spray structure of type Ib terminals (Figure 3). For instance, PNF is a type of flexibility training widely used to improve proprioception and treat chronic musculoskeletal pain, such as chronic neck and low back pain, knee osteoarthritis and lateral ankle sprain (Table 3). PNF involves both stretching and contracting the target muscle to achieve maximum flexibility and has been shown to improve the active and passive range of motion (Sharman et al., 2006). Furthermore, some exercises such as Tai‐Chi involve smooth and slow movements, which have been found beneficial for alleviating pain associated with fibromyalgia, knee osteoarthritis and chronic low back pain (Table 3) (Barnes et al., 2008). Nevertheless, further studies are needed to demonstrate whether exercise training can modulate proprioceptor activity and thus reset the hyperexcited proprioceptors to normal and treat pain.

**TABLE 3:** Pain management by proprioception‐associated training in various pain conditions.

Training	Pain
Proprioceptive neuromuscular facilitation	Chronic low back pain (8, 9, 10) Frozen shoulder (26) Knee osteoarthritis (27) Ankle sprain (31, 32) Subacromial impingement syndrome (37) Thumb carpometacarpal osteoarthritis (38) Thumb basal osteoarthritis (39)
Proprioceptive training	Chronic low back pain (19) Chronic neck pain (19, 23, 24, 25) Knee osteoarthritis (29) Lumbar radiculopathy (33) Painful epicondylitis (34) Thumb basal osteoarthritis (39)
Tai‐Chi	Fibromyalgia (1, 2, 3) Chronic low back pain (11, 12) Chronic neck pain (21) Knee osteoarthritis (28) Partial anterior cruciate ligament tears (35) Multiple sclerosis (11) Posttraumatic stress disorder (11)
Qi Gong	Fibromyalgia (4, 5)
Yoga	Fibromyalgia (6)
Core stability training	Chronic low back pain (12, 13, 14, 15, 16, 17)
Whole‐body vibration	Chronic low back pain (18)
Brahma mudra	Chronic neck pain (22)
Cranio‐cervical flexion	Chronic neck pain (23)
Balance exercise	Patellofemoral pain syndrome (36)
Walking	Knee osteoarthritis (30)
Aerobic exercise	Fibromyalgia (1)
Belly dance	Fibromyalgia (7)
Aquatic exercises in shallow water	Chronic low back pain (20)
References	
1: Wang et al. (2018), BMJ 360, k851, doi: 10.1136/bmj.k851	
2: Wang et al. (2010), N Engl J Med 363(8), 743–754, doi: 10.1056/NEJMoa0912611	
3: Jones et al. (2012), Clin Rheumatol 31(8), 1205–1214, doi: 10.1007/s10067‐012‐1996‐2	
4: Astin et al. (2003), J Rheumatol 30(10), 2257–2262, PMID: 14528526	
5: Liu et al. (2012), Int J Neurosci 122(11), 657–664, doi: 10.3109/00207454.2012.707713	
6: Carson et al. (2010), Pain 151(2), 530–539, doi: 10.1016/j.pain.2010.08.020	
7: Baptista et al. (2012), Clin Exp Rheumatol 30(6 Suppl 74), 18–23, PMID: 23020850	
8: Lakkadsha et al. (2022), Cureus 14(8), e27916, doi: 10.7759/cureus.27916	
9: Gao et al. (2022), J Back Musculoskelet Rehabil 35(1), 21–33, doi: 10.3233/BMR‐200306	
10: Arcanjo et al. (2022), Complement Ther Clin Pract 46, 101505, doi: 10.1016/j.ctcp.2021.101505	
11: Urits et al. (2021), Adv Ther 38(1), 76–89, doi: 10.1007/s12325‐020‐01554‐0	
12: Zou et al. (2019), Medicina 55(3), 60, doi: 10.3390/medicina55030060	
13: Kim et al. (2015), Clin Rehabil 29(7), 653–662, doi: 10.1177/0269215514552075	
14: Niederer and Mueller (2020), PLoS One 15(1), e0227423, doi: 10.1371/journal.pone.0227423	
15: Hlaing et al. (2021), BMC Musculoskelet Disord 22(1), 998, doi: 10.1186/s12891‐021‐04858‐6	
16: Lin et al. (2022), Int J Environ Res Public Health 19(6), 3324, doi: 10.3390/ijerph19063324	
17: Gorji et al. (2022), Int J Environ Res Public Health 19(5), 2694, doi: 10.3390/ijerph19052694	
18: Zheng et al. (2019), Med Sci Monit 25, 443–452, doi: 10.12659/MSM.912047	
19: McCaskey et al. (2014), BMC Musculoskelet Disord 15, 382, doi: 10.1186/1471‐2474‐15‐382	
20: Psycharakis et al. (2022), Physiotherapy 116, 108–118, doi: 10.1016/j.physio.2022.03.003	
21: Ross et al. (1999), J Holist Nurs 17(2), 139–147, doi: 10.1177/089801019901700203	
22: Jagadevan et al. (2921), J Bodyw Mov Ther 27, 717–722, doi: 10.1016/j.jbmt.2021.06.015	
23: Izquierdo et al. (2016), J Rehabil Med 48(1), 48–55, doi: 10.2340/16501977‐2034	
24: Revel et al. (1994), Arch Phys Med Rehabil 75(8), 895–899, doi: 10.1016/0003‐9993(94)90115‐5	
25: Espí‐López et al. (2021), Eur J Phys Rehabil Med 57(3), 397–405, doi: 10.23736/S1973‐9087.20.06302‐9	
26: Mertens et al. (2022), Rheumatol Int 42(6), 925–936, doi: 10.1007/s00296‐021‐04979‐0	
27: Shen et al. (2022), Am J Phys Med Rehabil 101(8), 753–760, doi: 10.1097/PHM.0000000000001906	
28: Kelley et al. (2022), Sci Prog 105(2), 368504221088375, doi: 10.1177/00368504221088375	
29: Jeong et al. (2019), J Athl Train 54(4), 418–428, doi: 10.4085/1062‐6050‐329‐17	
30: Goonasegaran et al. (2022), J Sports Med Phys Fitness 62(2), 229–237, doi: 10.23736/S0022‐4707.20.11686‐4	
31: Alahmari et al. (2020), Biomed Res Int 2020, 9012930, doi: 10.1155/2020/9012930	
32: Lazarou et al. (2018), J Back Musculoskelet Rehabil 31(3), 437–446, doi: 10.3233/BMR‐170836	
33: Senol et al. (2022), J Back Musculoskelet Rehabil 35(2), 421–428, doi: 10.3233/BMR‐200361	
34: Schiffke‐Juhász et al. (2021), J Orthop Surg Res 16(1), 468, doi: 10.1186/s13018‐021‐02602‐3	
35: Giummarra et al. (2022), BMC Musculoskelet Disord 23(1), 332, doi: 10.1186/s12891‐022‐05278‐w	
36: Lee et al. (2022), Medicine 101(37), e30631, doi: 10.1097/MD.0000000000030631	
37: İğrek and Çolak (2022), J Bodyw Mov Ther 30, 42–52, doi: 10.1016/j.jbmt.2021.10.015	
38: Campos‐Villegas et al. (2022), J Hand Ther S0894‐1130(22)00080‐1, doi: 10.1016/j.jht.2022.07.005	
39: Cantero‐Téllez et al. (2022), Int J Environ Res Public Health 19(6), 3592, doi: 10.3390/ijerph19063592	

### 4.2 A proprioceptor‐mediated unpleasant feeling

In 1996, Gillette and colleagues reported that patients with chronic muscle pain complained of a non‐nociceptive unpleasant feeling, possibly due to hyperactivity of proprioceptors (Kramis et al., 1996). Indeed, many patients with chronic muscle pain derived from nociceptor‐mediated pain cannot find relief from effective traditional pain killers such as non‐steroidal anti‐inflammatory drugs, corticosteroids and morphine. However, nociceptors may not (or only partially) contribute to this proprioceptive‐mediated unpleasant feeling. In 2018, from the study of the promiscuous properties of acid‐sensing somatosensory neurons, Chen and colleagues proposed a new concept of ‘sng’ to address the perception of acid sensation and to distinguish this concept from pain (Lin et al., 2018). This sng feeling is similar to the feeling of soreness after fatiguing exercise. It also occurs with massage treatment or successful acupuncture. Recently, sng has been found useful for characterizing the different phenotypes of fibromyalgia patients (Hsu et al., 2022; Hung et al., 2023). The metabolomic and proteomic profiles of sng‐dominant fibromyalgia patients differ substantially from those of pain‐dominant patients. Notably, the lactate level in blood and urine is significantly higher in sng‐ than pain‐dominant patients. With the acid‐sensing nature of proprioceptors, future studies could probe whether the proprioceptor‐mediated unpleasant feeling is sng or sng‐related and how it relates to pain management for intractable musculoskeletal pain such as fibromyalgia.

## 5 CONCLUSION AND FUTURE DIRECTION

In both clinical and animal studies, chronic musculoskeletal pain is highly related to proprioceptive abnormality and altered metabolism in muscle. Despite limited evidence supporting a role for proprioceptors in nociception, the roles of acid signalling in proprioception deserve further investigation. From molecular and physiological viewpoints, proprioceptors are sensitive to both acidosis and mechanical stimuli. Anatomical findings reveal that both nociceptor and proprioceptor nerve terminals are closely distributed in muscle spindles, and proprioceptor terminals can release glutamate to affect the nerve activities of both nociceptors and proprioceptors inside a muscle spindle capsule. Thus, the dual function of proprioceptors in sensing both extracellular pH and mechanical force suggests that they might play important roles in the development and maintenance of chronic musculoskeletal pain. Further studies with proprioceptor‐selective gene knockouts on proton‐sensing ion channels (e.g., ASIC1a, ASIC1b, ASIC2a, ASIC2a and ASIC3) would help reveal the roles of proprioceptors in acid‐related musculoskeletal pain in mouse models. Understanding the roles of proprioceptors in acid‐sensing properties and acid‐related pain would provide a new window to develop effective treatments for intractable chronic musculoskeletal pain.

## AUTHOR CONTRIBUTIONS

Cheng‐Han Lee prepared the figures, tables and structure of the manuscript and wrote the manuscript. Chih‐Cheng Chen prepared the structure of the manuscript and wrote the manuscript. All authors have read and approved the final version of this manuscript and agree to be accountable for all aspects of the work in ensuring that questions related to the accuracy or integrity of any part of the work are appropriately investigated and resolved. All persons designated as authors qualify for authorship, and all those who qualify for authorship are listed.

## CONFLICT OF INTEREST

No competing interests declared.
